# Innovative Approaches for Immune Tolerance to Factor VIII in the Treatment of Hemophilia A

**DOI:** 10.3389/fimmu.2017.01604

**Published:** 2017-11-24

**Authors:** Alexandra Sherman, Moanaro Biswas, Roland W. Herzog

**Affiliations:** ^1^Department of Pediatrics, University of Florida, Gainesville, FL, United States

**Keywords:** factor VIII, hemophilia A, immune tolerance, regulatory T cell, oral tolerance, rapamycin, gene therapy, AAV vectors

## Abstract

Hemophilia A (coagulation factor VIII deficiency) is a debilitating genetic disorder that is primarily treated with intravenous replacement therapy. Despite a variety of factor VIII protein formulations available, the risk of developing anti-dug antibodies (“inhibitors”) remains. Overall, 20–30% of patients with severe disease develop inhibitors. Current clinical immune tolerance induction protocols to eliminate inhibitors are not effective in all patients, and there are no prophylactic protocols to prevent the immune response. New experimental therapies, such as gene and cell therapies, show promising results in pre-clinical studies in animal models of hemophilia. Examples include hepatic gene transfer with viral vectors, genetically engineered regulatory T cells (Treg), *in vivo* Treg induction using immune modulatory drugs, and maternal antigen transfer. Furthermore, an oral tolerance protocol is being developed based on transgenic lettuce plants, which suppressed inhibitor formation in hemophilic mice and dogs. Hopefully, some of these innovative approaches will reduce the risk of and/or more effectively eliminate inhibitor formation in future treatment of hemophilia A.

## Introduction

Hemophilia A, or factor VIII (FVIII) deficiency, is one of the most prevalent genetic bleeding disorders, which affects 1:5,000 male births. It is inherited as an X-linked recessive trait, although it can also be acquired in advanced age as a result of autoimmunity, cancer, or various metabolic disorders affecting both males and females (1). Both inherited and acquired forms of hemophilia are manifested by frequent bleeding episodes, hemorrhages into the skin and body cavities, pain, orthopedic disability, and significant morbidity (2).

Classification and severity of hemophilia has been based on circulating levels and residual activity of coagulation factors in plasma. Residual factor activity levels of <1%, compared to normal plasma are classified as severe, 1–5% moderate, and 5–40% mild (3). Patients with large deletions in the F8 gene, or inversion mutation in intron 22 (I22I), have no circulating FVIII, suffer from severe hemophilia and are most likely to develop an adverse immune reaction to exogenous FVIII infusions (4). Patients with a missense or disruption mutation may express various amounts of non-functional or partially functional FVIII protein. Patients with milder forms of hemophilia A respond better to treatment and are at a lower risk of developing adverse inhibitory antibodies (5).

Factor VIII is a 280-kDa glycoprotein that circulates in the blood at ~200 ng/ml (where it is closely associated with von Willebrand factor) and, upon activation, serves as a co-factor to the serine protease factor IX (FIX), which catalyzes a critical step in the intrinsic pathway of the coagulation cascade. FVIII is initially synthesized as a 2,351-aa polypeptide that is organized into A1–A2–B–A3–C1–C2 domains and processed into non-covalently linked heavy and light chains prior to secretion. Since FVIII is critical for the enzymatic function of FIX, mutations in either protein can cause the bleeding phenotype that is characteristic for hemophilia. The majority of hemophilic patients have mutations in their F8 gene (resulting in hemophilia A), while mutations in F9 result in hemophilia B.

It is estimated that up to 30% of patients with severe hemophilia A and 5% of patients with milder forms of the disease form anti-drug antibodies, termed “inhibitors.” These inhibitors are detected and measured in the Bethesda assay, with 1 Bethesda unit representing 50% residual coagulation activity in normal plasma after incubation with a patient’s test plasma. That fact that potent antibody responses occur despite FVIII being given intravenously at low antigen doses illustrates the immunogenicity of this protein. Patients with >5 BU/ml typically fail to respond to factor replacement therapy, requiring the use of bypass agents. Recent clinical studies have demonstrated that genetic variables and F8 gene mutation type are important determinants of an individual’s risk for inhibitor formation, as is ethnicity and intensity of early treatment (6). While clinical protocols have been available to restore hemostasis in inhibitor patients and to reverse the inhibitor response, these methods are suboptimal, expensive, and not successful in all patients. Moreover, there are no prophylactic protocols to prevent inhibitor formation. These limitations have fueled recent diverse pre-clinical developments of alternative strategies for immune tolerance induction (ITI) to FVIII, which are based on emerging technologies such as gene therapy, regulatory T-cell (Treg) therapy, and transgenic crop plants for oral tolerance, among others (7).

## Current and Future Treatments of Hemophilia and Their Immune Implications

Currently, hemophilia A is treated with an intravenous infusion of plasma-derived or recombinant clotting FVIII concentrates, which can be on demand or prophylactic (8, 9). However, the half-life of infused FVIII concentrate is very short, only 10–12 h, and thus, it must be administered frequently, an inconvenience for the patient. Also, these frequent infusions create the possibility of introducing infections through the indwelling catheter as well as a risk of micro bleeds. Therefore, the development of longer acting FVIII concentrates became the next step in clinical care for HA patients (10). Longer lasting, or extended half-life clotting factors have been recently introduced for therapeutic and prophylactic treatment of hemophilia A. These include Fc and albumin fusion proteins as well as PEGylated FVIII (11–15). However, half-life extension of these products has been modest (more than twofold increase) (10, 16). Based on older observations on tolerogenic effects of immunoglobulin conjugation, it is hoped that Fc-FVIII may have reduced immunogenicity, which is supported by some pre-clinical data (17). Clinical evaluation in previously untreated patients (PUPs) should answer this question.

As opposed to generating less immunogenic FVIII molecules or employing ITI, an alternative strategy to avoid the effects of inhibitors against FVIII altogether is to develop bypassing agents that promote coagulation through pathways that either do not require FVIII or that mimic the function of FVIII. Novel drugs that fall into this category include Emicizumab (Chugai Pharmaceutical, Chuo, Tokyo, Japan), a human monoclonal bi-specific antibody, which is administered subcutaneously once per week (18, 19) and binds to both activated coagulation FIX and FX, mimicking the function of FVIII (20). Fitusiran is an experimental RNAi-based drug developed by Alnylam Pharmaceuticals (Cambridge, MA, USA) that targets endogenous anticoagulant antithrombin expression in the liver (21). As a result, Fitusiran improves homeostasis by promoting thrombin generation. Both drugs are currently undergoing extensive clinical testing.

Rather than treating hemophilia with more or less frequent drug administrations, gene therapy has the potential to cure the disease. Multiple Phase I/II clinical trials are testing hepatic *in vivo* gene transfer with adeno-associated viral (AAV) vectors in patients with severe hemophilia A, in some cases achieving normal FVIII levels (22). In pre-clinical large animal studies, sustained expression for >1 decade had been observed with this approach. While FVIII is normally produced by liver endothelial cells, these gene therapies target transgene expression to the more abundant hepatocytes. In these trials, patients must have demonstrated extensive prior treatment with FVIII protein without having formed inhibitors. Nonetheless, a large body of studies in animal models of hemophilia has demonstrated the potential of hepatic gene transfer to induce immune tolerance to the transgene product, which is discussed in further detail below.

## Inhibitor Formation and Clinical ITI

In traditional intravenous FVIII replacement therapy, the appearance of inhibitors is usually observed in PUPs, i.e., young pediatric patients, during the first 50 days of exposure to FVIII (23). However, increased incidence of inhibitor development was also reported in older patients (50+ years), with previous exposure to FVIII (24). Inhibitor formation is a serious complication in the treatment of hemophilia. These antibodies make replacement therapy ineffective, thereby substantially complicating treatment, increase risks of morbidity and mortality, and substantially elevate costs of treatment. The mechanism of inhibitor formation is multifactorial and not entirely understood. Several predisposing risk factors have been identified. Genetic risk factors include F8 mutation types (such as large deletions, nonsense mutations, and intron 22 inversions), which are associated with a higher rate of inhibitor development. Patients of African-American and Hispanic ethnicity have a higher risk for inhibitor formation. Family and sibling history, major histocompatibility complex class II alleles, and polymorphisms in immune regulatory genes coding for cytokines (IL-10, TNFα) and other molecules such as CTLA-4 are likely important contributors (25, 26). Other modifiers include production of indoleamine-pyrrole 2,3-dioxygenase (IDO) enzyme, inflammation, and age and intensity of first exposure to FVIII (27, 28).

B-cell activation, leading to inhibitor formation, is CD4^+^ T-helper cell dependent, and several CD4^+^ T-cell epitopes have been mapped in humans (29). Co-stimulation via CD80/86-CD28, CD40-40L, and ICOS-ICOSL pathways is required, which can be exploited for tolerance induction using co-stimulation blockers such as anti-CD40L/CTLA-4-IgG combination or anti-ICOS monoclonal antibody (30). A related strategy is based on interference with T-cell receptor (TCR) signaling using anti-CD3, which appears to favor induction of CD4^+^CD25^+^FoxP3^+^ Tregs (31). Inhibitors target various parts of FVIII, although the A2 and C2 domains are believed to be the most immunogenic. Marginal zone macrophages have been found to be important for the capture and accumulation of FVIII in the spleen (32). Otherwise, remarkably little is known about the roles of professional antigen-presenting cells (APCs), such as dendritic cells (DCs) in the events that lead to FVIII-specific B-cell activation. Memory T cells may be re-activated by innate immune signaling through toll-like receptors 7 or 9 (33, 34).

**Figure 1:**
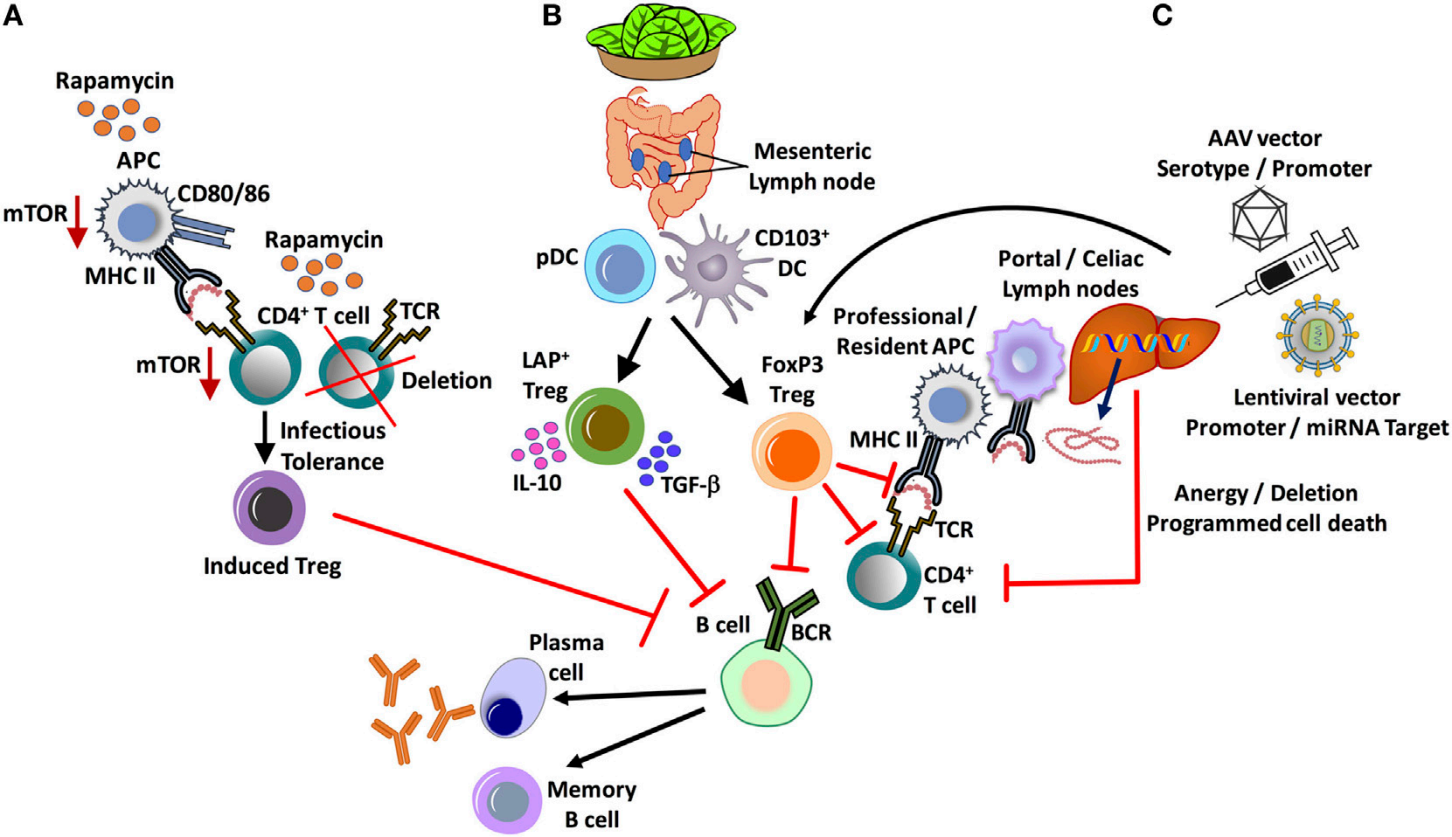
Major *in vivo* approaches for tolerance induction and suppression of inhibitor formation in hemophilia. **(A)** Administration of the widely used clinical immunosuppressant, rapamycin, results in the selective deletion of CD4^+^ T-helper cells and enrichment of FoxP3^+^ regulatory T cell (Treg), exploiting differential use of the mTOR pathway. **(B)** Oral tolerance by lettuce encapsulated clotting factor leads to the suppression of inhibitors by at least two subsets of Treg: CD4^+^CD25^−^LAP^+^FoxP3^−^ and CD4^+^CD25^+^FoxP3^+^ Treg. Antigen presentation by gut resident CD103^+^ dendritic cell (DC), as well as plasmacytoid DCs (pDCs), supports Treg induction. **(C)** Hepatic gene transfer by adeno-associated viral (AAV) or lentiviral vector induces tolerance by multiple mechanisms, which include programmed cell death of CD4^+^ T-helper cells and the induction of FoxP3^+^ Treg. An important role for initial antigen presentation in the liver draining portal/celiac lymph nodes by professional as well as liver resident antigen-presenting cells (APCs) is indicated.

Current clinical protocols for ITI are designed to eradicate inhibitors. The most commonly used form of ITI employs frequent (daily), high dose (up to 200 IU/kg/day) infusions of FVIII to eliminate inhibitors (35, 36). To date, there is no definitive mechanistic explanation as to how high doses of FVIII can induce tolerance. One of the proposed theories is that repetitive, high doses of antigen can suppress activated T-cell responses by overstimulation with antigen, followed by anergy and deletion (37). ITI also targets FVIII-specific memory cells and may assist in the induction of Treg (38). ITI is considered successful if inhibitor titers fall below 0.6 BU/ml, and FVIII function is normalized (39). Duration of ITI varies among patients from 9 to 48 months, according to the International Immune Tolerance Registry and the North American Immune Tolerance Registry. Therefore, ITI protocols often cost >$1M to complete. Outcomes of ITI therapies are variable as well. Only 50–70% of patients benefit from “traditional” ITI protocols. Some patients, who initially respond to ITI therapy, may experience anamnesis (inhibitor re-appearance) with repeated exposure to FVIII. Taking in consideration the high cost, moderate success rate, long duration, inconvenience of daily infusions, and a risk of anamnesis, ITI protocols can be modified to include other therapies and immunomodulation (Figure 1).

## Novel Approaches to Immune Tolerance in Hemophilia—*in vivo* Treg Induction Vs Treg Therapy

Over the past decade, strong evidence has emerged that Tregs are an integral part of immune tolerance to coagulation factors in gene and protein replacement therapies (40–42). It should therefore be possible to promote tolerance to FVIII by enhancing *in vivo* Treg induction or by development of a Treg-based cell therapy. Thymic-derived and peripherally induced CD4^+^CD25^+^FoxP3^+^ Tregs are critical immune regulators to prevent autoimmune disease and for tolerance to “non-self” antigens. Treg may suppress immune responses via diverse mechanisms that include direct cell to cell contact, release of suppressive cytokine such as IL-10 and/or TGF-β cytokines, and modulation of APC maturation and function, thus preventing differentiation of T cells into effector cells and promoting their conversion into Tregs (43). One potent approach to shifting the balance from an effector T-cell to a Treg response to an exogenous protein is co-administration of the antigen with the mTOR inhibitor rapamycin (44) (Figure 1).

Cell cycle progression in activated T cells upon stimulation of the IL-2 receptor requires signaling through the mTOR pathway in conventional T cells. Activation of mTOR also promotes glycolysis, a metabolic pathway that effector T cells heavily depend on. Therefore, antigen presentation combined with blockage of the mTOR pathway by rapamycin results in apoptosis of activated T cells. However, Treg induction is enhanced in the presence of rapamycin. Treg heavily depend on IL-2 but preferentially utilize alternative downstream signaling pathways (via Stat5) and lipid metabolism, allowing these cells to expand in the presence of rapamycin. Hence, a 1-month oral regimen of rapamycin, combined with low-dose intravenous FVIII administration, resulted in long-term tolerance to therapeutic FVIII administration in hemophilia A mice (45). Antigen-specific tolerance was maintained for months after general immune suppressive effects had waned. This outcome correlated with the induction of FVIII-specific CD4^+^CD25^+^FoxP3^+^ Treg. To further enhance efficacy and reduce systemic immune suppressive effects, rapamycin may be packaged into polymeric synthetic nanoparticles. Transient co-administration of FVIII and rapamycin-nanoparticles similarly induced lasting tolerance in the hemophilia A mouse model and also diminished pre-existing inhibitors (46, 47). The tolerogenic effect of rapamycin can be further enhanced by addition of cytokines such as IL-10 or Flt3L (44, 45, 48). Flt3L is widely used for *in vivo* expansion of DCs. Interestingly, in the presence of rapamycin (within a certain dose range), Flt3L selectively expands plasmacytoid DCs (pDCs), resulting in further increased Treg induction compared to antigen/rapamycin alone (48). In contrast to other DCs, pDCs uniquely express a microRNA (miRNA) that indirectly causes a more active mTOR pathway (48, 49). Hence, pDCs are more resistant to mTOR inhibition. Evidence has been presented that pDC enhances Treg induction through expression of IDO, which has effects on signal transduction but also catalyzes the first step in tryptophan catabolism, resulting in degradation products that may promote Treg induction (50).

An alternative method to enrich for Treg *in vivo* is the use of IL-2 complexed with a monoclonal antibody against IL-2 (IL-2/IL-2 mAb complexes), thereby aiding in the rapid expansion of CD4^+^CD25^+^FoxP3^+^ Treg. Pretreatment with these complexes have been shown to produce activated and highly suppressive Treg in mice that prevented autoimmunity and showed long-term acceptance in transplant rejection studies (51). In hemophilia A mice, this regimen robustly suppressed inhibitor formation to either FVIII replacement therapy or plasmid-mediated gene therapy of FVIII. Long-term tolerance to FVIII, which resisted subsequent re-challenge with FVIII protein, was observed and was attributed to TGF-β1-dependent conversion of FVIII-specific CD4^+^CD25^−^ conventional T cells into Treg (52, 53).

**Figure 2:**
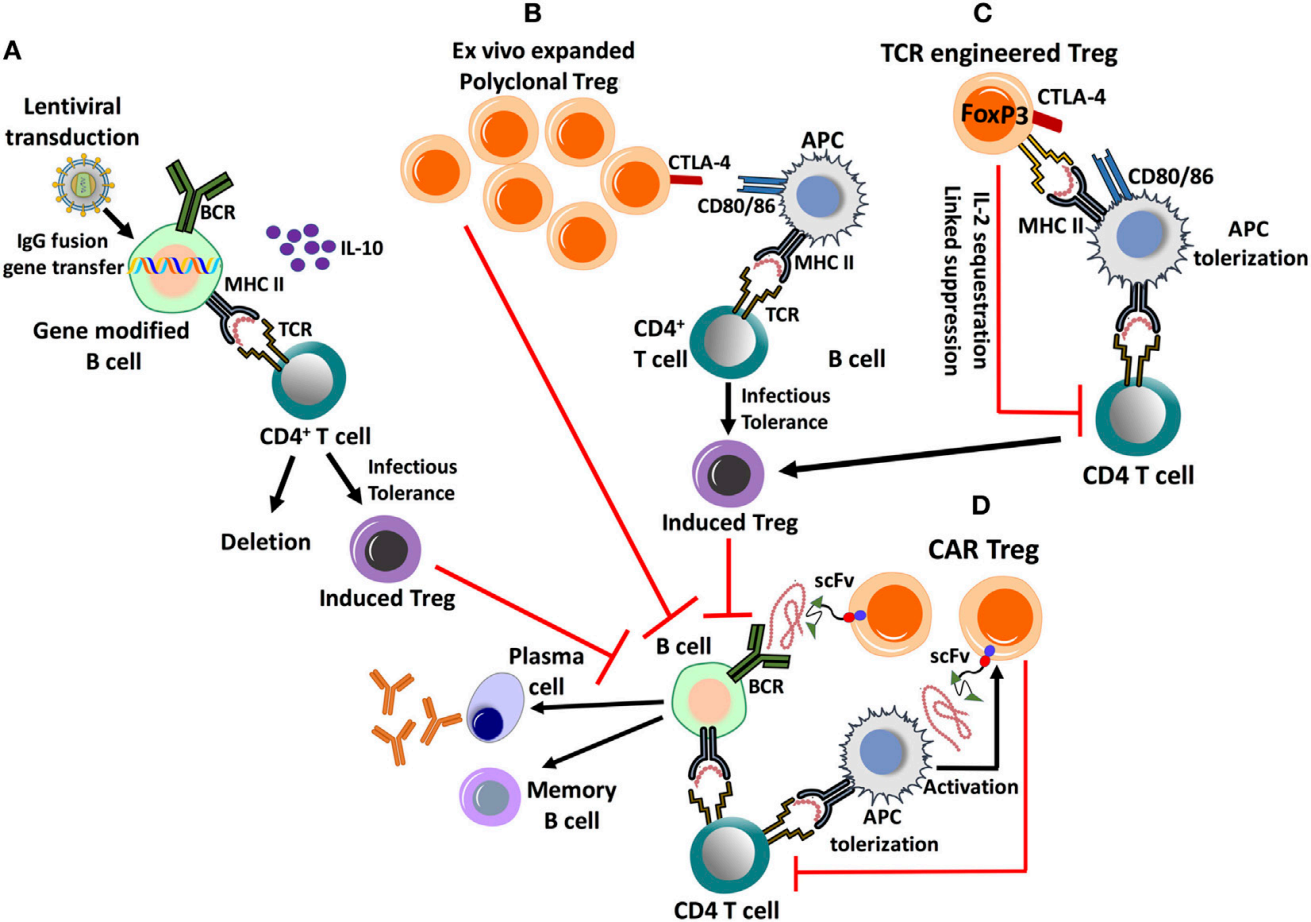
Emerging cell therapy-based approaches to eliminate inhibitor formation. These proposed treatments are based on the *ex vivo* expansion/engineering of autologous lymphocytes, followed by adoptive transfer back into the patient. **(A)** Lentivirally transduced, gene modified B cells expressing an IgG fusion protein can induce tolerance by MHC II presentation of the clotting factor product, which results in the deletion of effector CD4^+^ T cells and induction of CD4^+^CD25^+^FoxP3^+^ regulatory T cell (Treg). Tolerance induction is dependent on IL-10 production. **(B)** *Ex vivo* expanded, polyclonal CD4^+^CD25^+^FoxP3^+^ Treg highly up-regulate CTLA-4, promoting antigen-presenting cell (APC) tolerization by binding to co-stimulatory CD80/86 molecules. This facilitates the conversion of CD4^+^ T helper cells into induced Treg by a process of infectious tolerance, subsequently leading to antigen-specific suppression. **(C)** Treg can be engineered to express a factor VIII-specific T-cell receptor (TCR), redirecting antigen recognition to a specific, MHC II-restricted epitope of the clotting factor. They can suppress CD4^+^ T-cell and B-cell responses by multiple mechanisms. **(D)** Chimeric antigen receptor (CAR) Treg is engineered by introducing antigen recognizing single-chain variable fragment (scFv) antibody domains, fused to primary and co-stimulatory TCR signaling molecules. CAR Treg can recognize clotting factor bound to the surface of APC, leading to their activation and suppressive mechanisms, which include APC tolerization and CD4^+^ T-cell inhibition. It is yet unknown whether CAR Treg can directly suppress antigen-specific B cells.

A different approach to utilizing Treg to suppress inhibitor formation is that of a cell therapy (Figure 2). For example, *ex vivo* expanded polyclonal CD4^+^CD25^+^FoxP3^+^ Tregs are successfully used in hematopoietic stem cell transplants to prevent graft vs host disease are also evaluated in the treatment of autoimmune disease (54). Methods for the expansion of clinical-grade human Treg are well established and continuously further optimized (55). *Ex vivo* expanded Tregs highly up-regulate CTLA-4, enabling them to down-regulate the co-stimulatory signaling molecules CD80/CD86 upon interaction with DCs, thereby promoting tolerogenic antigen presentation (56, 57) (Figure 2). In the hemophilia A mouse, transplant of polyclonal Tregs, which had been *ex vivo* expanded with anti-CD3/-anti-CD28 beads and IL-2, suppressed inhibitor formation against FVIII protein therapy even after the transferred cells become undetectable (56). Adoptive transfer and *in vitro* studies revealed the ability of the expanded non-specific Treg to enhance induction of endogenous, antigen-specific Treg by facilitating conversion of conventional specific CD4^+^ T cells to CD4^+^CD25^+^FoxP3^+^ Treg (56) (Figure 2). This approach has the advantages of availability of clinical protocols and reagents and of not requiring genetic manipulation of patient cells. However, large cell numbers are likely required, and therefore, there is a risk of general immune suppression early after cell transplant (Table 1).

**Table 1:** Summary of main approaches currently being developed for tolerance induction to factor VIII (FVIII).

Approach	Mechanism	Advantages	Disadvantages
Hepatic gene transfer	Induction of FoxP3^+^ regulatory T cells (Tregs) and deletion of effector T cells	Combines treatment with immune tolerance induction; potential for inhibitor reversal; already in advanced clinical development as a therapy for adults	Requires gene transfer to pediatric patients; immune responses to viral vectors have been observed clinically

Co-administration of FVIII with rapamycin (potentially combined with cytokines)	*In vivo* induction of FoxP3^+^ Treg combined with deletion of effector T cells by inhibition of mTOR pathway	Lasting tolerance induction after transient regiment	Transient general immunosuppression

*Ex vivo* expansion of polyclonal Treg	Down-regulation of co-stimulatory molecules CD80/CD86, promoting tolerogenic antigen presentation and endogenous Treg induction	Clinical protocols already established	Transient immune suppressive effects/initial lack of antigen-specificity, large number of cells required

*Ex vivo* gene-modified Tregs	FVIII-specific suppression by FoxP3^+^ Treg with specificity redirected by T-cell receptor (TCR) or chimeric antigen receptor (CAR) gene transfer	Reduced cell numbers for therapy, antigen-specificity, no MHC restrictions for CAR approach	Genetic manipulation of patient cells required, MHC restriction for the TCR approach, durability, and costs unclear

*Ex vivo* gene modified B cells	Treg induction and effector T-cell deletion via MHC II presentation by transduced B cells	Highly effective in animal models	Use of integrating vectors required, large number of cells required, limitations to titers of current lentiviral vectors that transduced human B cells

B-cell depletion with rituximab (anti-CD20)	Depletion of CD20^+^ B cells	Reduces inhibitors in some patients that failed traditional immune tolerance induction, can potentially be combined with rapamycin to induce tolerance in such patients	Does not target plasma cells, inhibitors tend to relapse (although the outcome may be improved by combination with other drugs such as rapamycin)

Oral tolerance	Bioencapsulation and targeting of antigen to immune system of small intestine, induction of FoxP3^+^ and LAP^+^ Tregs	Could be considered prophylactically, antigen-specific tolerance without immunosuppression or genetic manipulation, low production cost	Repeat oral delivery appears required for lasting tolerance

Hence, efforts are directed at generation of FVIII-specific Treg. This can be accomplished by redirecting antigen-specificity through TCR or chimeric antigen receptor (CAR) gene transfer to Treg (Figure 2). FVIII-specific Tregs generated by engineering Tregs to express a single human TCR (upon *ex vivo* retroviral gene transfer) have been found to suppress CD4^+^ T-cell and B-cell responses and to be suppressive in hemophilia A mice expressing human HLA (58). Although these Tregs only recognized a single epitope, suppression of responses against the entire FVIII molecule occurred. Nonetheless, because of differences in HLA, translation of this strategy would require cloning of multiple TCRs. In contrast to transferring a TCR, the CAR approach is that it is not MHC restricted. Therefore, one construct could theoretically be used for all patients. CAR T cells are engineered by introducing antigen recognizing variable region (single-chain variable fragment) antibody domains, fused to primary and co-stimulatory signaling molecules. Antigen recognition and cell signaling by CAR expressing T lymphocytes are therefore independent of APCs and is not MHC restricted (59, 60). Successful trials of CAR T-cell immunotherapies for the treatment of leukemia have created possibilities of engineering FVIII-specific CAR Tregs, with antigen-specific suppression (61, 62). Experimentally generated FVIII-specific human CAR Tregs suppressed antibody formation *in vitro* and *in vivo* in hemophilia A mice (58, 63). CAR T cells typically recognize antigens of cell surfaces. The mechanism of suppression of responses to a soluble protein such as FVIII is, therefore, not entirely clear. For *in vitro* suppression, APCs were required, suggesting that cell surface association is needed (Figure 2). A related question is whether CAR Treg may be able to directly suppress B cells. Prior to translation of the approach, questions regarding the *in vivo* persistence, durability of suppression, and safety need to be addressed.

## Directly Targeting B Cells for Tolerance

Upon activation, B cells differentiate into memory cells or antibody secreting cells, including plasma cells. However, B cells also play a role in antigen presentation (64). Interestingly, gene-modified primary B cells have the capacity to induce immune tolerance upon retroviral or lentiviral gene transfer (while TLR9 activation during plasmid gene transfer generates immunogenic B cells) (65–67). Skupsky et al. showed that the expression of IgG fusion proteins (IgG-A2 and IgG-C2 domains of FVIII) in primary B cells is a particularly powerful tool to induce tolerance (65). Gene-modified B cells route the expressed fusion protein through the endosomal compartment, resulting in MHC II presentation, deletion of effector T cells, and induction of CD4^+^CD25^+^FoxP3^+^ Treg (Figure 2) (68). Adoptive transfer of a mixture of retrovirally transduced B cells, expressing IgG fusion of FVIII A2 or C2 domain, suppressed inhibitor formation in hemophilia A mice (69). Similarly, IgG-F9 gene transfer prevented and reversed inhibitor formation and anaphylaxis against FIX in mice with hemophilia B (FIX deficiency) (66). A major limitation of this approach had been a lack of suitable gene transfer vectors for human B cells. Recent development of lentiviral vectors (LV) targeted to human CD20 through inclusion of a single chain antibody fragment in the viral envelope protein has overcome this bottle neck (67). Primary B cells transduced with such a LV to express IgG-FIX prevented inhibitor formation in hemophilia B mice (67). However, clinical translation is still not straightforward because of relatively low titers of this vector.

Theoretically, protocols directed at elimination of memory B cells should benefit patients with persistent inhibitors, in particular those who fail to respond to traditional ITI. Hence use of the monoclonal antibody rituximab, which eliminated CD20^+^ cells in the human body through a variety of mechanisms, has been tested for this purpose (70). A recent Phase II safety study demonstrated that the anti-CD20 antibody rituximab showed a modest affect in reducing inhibitors and preventing anamnestic responses to repeated exposures to FVIII, albeit relapse of the inhibitor response remains a problem (71). B-cell depletion by itself may not be effective for tolerance induction, which likely requires tolerance induction in the T-cell compartment. Therefore, combination therapy with additional drugs may be needed. Interestingly, a recent study showed that combining anti-CD20-mediated B-cell deletion with rapamycin and FVIII antigen substantially improved reversal of inhibitor formation in hemophilia A mice (72). Such a protocol was superior to anti-CD20 or rapamycin alone or to polyclonal Treg therapy. B-cell depletion was also shown to enhance tolerance induction to FVIII in the context of hepatic gene transfer, when transgene expression was low and thus ineffective in Treg induction (73).

## Hepatic Gene Therapy for Tolerance Induction

In contrast to protein replacement therapy, gene therapy has the potential for a lasting cure of hemophilia. A first successful gene therapy for hemophilia B, utilizing *in vivo* gene transfer to the liver with an AAV vector, was documented in recent years (74). Currently, there are multiple clinical trials using liver-directed AAV vectors to treat hemophilia A and B (75). Some of these are achieving levels of FVIII or FIX activity at or near normal, and are thus expected to progress to Phase III trials (76). AAV vectors are comprised of a DNA genome (that is either single-stranded or modified to be self-complementary) packaged into a protein capsid. These vectors, derived from a small non-pathogenic parvovirus, lack viral coding sequences and effectively transfer genes *in vivo*. Viral capsids with a tropism for the liver are utilized in current trials, and the therapeutic gene is under transcriptional control of a hepatocyte-specific promoter. Given the limited packaging capacity of the vector (~5 kb), B domain-deleted FVIII (BDD-FVIII) is expressed. The B domain is dispensable for FVIII activity, and several recombinant FVIII products are BDD. The liver is an ideal target organ for gene therapy for hemophilia. FVIII and FIX are normally synthetized by liver sinusoidal endothelial cells (LSECs) and hepatocytes, respectively (77). Both cell types can efficiently secrete proteins into circulation.

From an immunological point of view, hepatic gene transfer has the major advantage that transgene expression in the hepatic environment can induce immune tolerance (78) (Figure 1). Given their hepatotropism, low innate immunity (resulting in limited tissue inflammation at the time of gene transfer), and the inefficiency in transducing professional APCs, AAV vectors derived from several serotypes are ideal to achieve immune tolerance by hepatic gene transfer. Higher expression levels, as determined by vector serotype, dose, and the transgene expression cassette, favor tolerance induction (61, 79). High expression of the antigen enhances Treg induction and direct inhibition of memory B cells (80).

Constitutive exposure to FIX by hepatic gene transfer has been associated with CD4^+^ and CD8^+^ T-cell unresponsiveness and deletion by programmed cell death using both extrinsic and intrinsic mechanisms (79, 81, 82). In mice injected with AAV expressing the model antigen ovalbumin (AAV8-OVA), transgene-specific CD8^+^ T cells transiently up-regulated negative checkpoint markers, e.g., the programmed death 1 receptor, leading to inefficient killing of transduced hepatocytes. Tolerance induction to FVIII or FIX has been shown to rely on the induction and enrichment of CD4^+^CD25^+^FoxP3^+^ Tregs, which suppressed CD8^+^ T cells and antibody formation in both mice and non-human primates (79, 83–85). Studies in Fas-deficient mice suggested that Treg induction and T-cell deletion were both required for robust tolerance induction (79, 84). Intrahepatic IL-10 expression further enforced suppression of CD8^+^ T cells, without affecting antibody levels, while TGF-β expression was required for both Treg induction and to control transgene antibody formation in AAV-hFIX transduced hepatocytes (86). Engagement of the transmembrane protein GITR enhanced the proliferation and suppressive capacity of induced Tregs (84, 87, 88).

Antigen presentation in the tolerogenic liver environment by both professional APCs and liver resident cells is not fully understood but orchestrates a balance between immune regulation and immune surveillance (89). Administration of hepatotropic AAV8-OVA identified CD11c^+^ DC and macrophages as APCs that are required for MHC II presentation of the transgene product, which primarily occurs in liver draining lymph nodes, such as the portal and celiac lymph nodes, although the liver itself may also contribute to Treg induction (Figure 1) (90). Liver-induced Treg rapidly disseminated through the circulation into multiple lymphoid organs, which resulted in systemic regulation of the response to the AAV gene product.

There have been concerns regarding the potential for cellular stress in the liver by over-expression of FVIII in hepatocytes. Improvements such as codon optimization of the F8 gene and deletion of the B domain have, however, resulted in only a mild activation of the unfolded protein response in mice, which did not impact liver pathology or FVIII immunogenicity (91, 92). Codon optimization of the F8 gene yielded higher hepatic expression levels that sustained therapeutic expression and improved tolerance induction (73). Translation of these studies in a large animal model for hemophilia A showed that AAV-mediated liver gene transfer of canine FVIII was not only effective in long-term sustained expression of FVIII but may also eradicate pre-existing inhibitory antibodies in two strains of hemophilia A dogs, with indications for improving outcomes in patients with established inhibitors (93–95).

The capacity of liver-directed gene transfer to induce immune tolerance to transgene products has also been demonstrated for LV. The large gene carrying capacity of LV makes them attractive candidates for hemophilia A gene therapy. Improved safety profiles have been reported with the development of hepatocyte-targeted, integrase-defective LV, which resulted in a sustained expression of FIX, tolerant to neutralizing antibody induction in hemophilia B mice, and without the risk of insertional mutagenesis (96). LV more efficiently transduce a variety of APCs, leading to innate immune responses, including TLR7 and TLR9 activation, and the induction of type I interferon and pro-inflammatory cytokines (97, 98). Ultimately, this immune activation drives CD8^+^ T-cell and antibody responses against the transgene product. Transcriptional and post-translational engineering of the LV, using a combination of cell-specific promoters and miRNA target sequences to eliminate transgene expression in professional APCs (miR-142-3p), while restricting high levels of therapeutic expression to hepatocytes, has been shown to induce tolerance in both hemophilia A and B models and correction of disease phenotype (99–102).

A recent study has shown that directing LV-mediated FVIII gene expression to LSECs (which are the physiological source of FVIII synthesis) by using an endothelial cell-specific promoter, similarly resulted in stable and therapeutic levels of FVIII in mice (103, 104). Interestingly, using a CD11b myeloid cell-specific promoter and a target sequence for miR-126, which is highly expressed in endothelial and pDCs, resulted in the prevention of inhibitory antibodies to FVIII. Even after subsequent challenge of these mice with FVIII in adjuvant, they remained tolerant for up to 24 weeks. Therefore, an important contribution of gene transfer to pDCs in driving an immune to the FVIII antigen in LV gene therapy was proposed.

## Oral Tolerance Induction Using Transgenic Crop Plants

The immune system of the small intestine has evolved to promote tolerance to food antigens (105, 106). This phenomenon can be exploited in tolerance induction through oral antigen delivery. This concept has multiple advantages, since no immune suppressive drugs, genetic manipulation of host cells, or expensive cell therapies are required. Oral tolerance has been studied for more than half a century and is defined as a systemic immunological unresponsiveness or hyporesponsiveness to an orally administered antigen. Several recent successes in prevention of food allergies illustrate relevance for human treatment (107, 108). In experimental models of autoimmune diseases, orally administered antigens suppressed autoimmunity in animal models of experimental autoimmune encephalomyelitis, diabetes, and rheumatoid arthritis (109–111).

The mammalian digestive system has a rich and complex immune network that has evolved to maintain a delicate balance between tolerance and immunoreactivity (112, 113). The gut-associated lymphoid tissue consists of intestinal epithelial lymphocytes, concentrated within the intestinal epithelial barrier, Peyer’s patches, and mesenteric lymph nodes (MLNs) (114, 115). The majority of the incoming food proteins get digested and degraded in the stomach and upper intestine. Proteins that escape degradation pass through the gut epithelial barrier and are sampled by APCs. Antigen loaded APCs subsequently migrate to the MLNs (116, 117), where APCs activate and prime naïve antigen-specific T lymphocytes (118). Lillicrap and colleagues initially tested this mucosal tolerance concept for treatment of hemophilia A. Mice exposed to the immunogenic C2 domain of FVIII (FVIII-C2) via oral or nasal route developed partial tolerance to systemic challenges with FVIII-C2 and full-length FVIII. This tolerance persisted after adoptive transfer of CD4^+^ splenocytes from FVIII-KO mice that received intranasal antigen administration (119).

However, for the concept to go forward, one had to develop a technology for cost effective production of the FVIII antigen, protection from degradation in the stomach, and effective delivery to the gut immune system. Answers to these challenges came with advancements in plant genetics, resulting in high levels of expression of human therapeutic proteins in the chloroplast (120, 121) of crop plants for the production of edible biopharmaceuticals (122). Initial experiments conducted in hemophilia B mice using frozen and ground tobacco leaves demonstrated robust suppression of inhibitor formation and of fatal anaphylactic reactions against intravenous FIX (123). In subsequent studies in hemophilia A mice, using a mixture of frozen tobacco leaves expressing either C2 domain or the heavy chain of human BDD-FVIII, effective suppression of inhibitor formation was documented in two different strains of hemophilia A mice (124). In both studies, the bioencapsulated antigens were given by oral gavage twice per week, starting 1 month prior to initiation of traditional replacement therapy. This method could also reverse FIX inhibitors and desensitize from the allergic reactions to FIX in hemophilia B mice, as well as accelerate the decline of pre-existing FVIII inhibitors in hemophilia A mice (124, 125).

Toward an oral tolerance protocol that is feasible in humans, transgenic lettuce plants were developed. This became feasible after identification of lettuce chloroplast-specific posttranscriptional elements that ensure high expression (126, 127). Furthermore, chloroplast genomics tools were developed for the identification of ribosomal stall sites and optimization of codon usage (128). In addition, growth of the transplastomic lettuce in a hydroponic system suitable for GMP production was developed, as well as a lyophilization process to generate leaf material for stable long-term storage at ambient temperature. When tested in hemophilia B mice, lyophilized lettuce containing human FIX was effective in tolerance induction over a wide range of antigen doses (127). To prove that the method is not limited to rodent models, a study in hemophilia B dogs was performed. These animals are similar in size to pediatric patients and reproducibly form antibodies against human FIX after repeated intravenous delivery. Three of the four dogs that received the oral tolerance regimen at an antigen dose of 0.3 mg/kg showed robust suppression in IgG and IgE formation against human FIX, correlating with a lack of inhibitor formation, lack of anaphylactic reactions, and restoration of blood clotting times after each of 8 weekly FIX injections (129). Extensive serum chemistry, hematological, and general health evaluations showed absence of toxicity even after several months of oral delivery.

The mechanism of plant induced oral tolerance is complex. The plant cell wall provides bioencapsulation for the antigens, which are released in the small intestine through degradation by enzymes produced by intestinal microbes. For efficient delivery across the gut epithelium, FVIII and FIX antigens are expressed in chloroplasts as N-terminal fusions to cholera toxin B (CTB) subunit. CTB is an effective transmucosal carrier that, in the form of a pentamer, binds to the GM1 receptor on the surface of epithelial cells (and other cell types, including DCs), resulting in uptake and translocation through transcytosis/retrograde trafficking (130). Inclusion of a furin cleavage sites assures release of FVIII or FIX sequence from CTB. Immunohistochemistry showed delivery to DCs in the lamina propria and in Peyer’s patches (125). These include CD103^+^ DC (Figure 1). Upon intravenous challenge with antigen, increases in the frequencies of CD103^+^ DC and pDCs are observed, especially in MLNs. CD103^+^ DC are critical APCs in oral tolerance induction, since they transport antigen to the MLN, where they effectively induce Treg. The plant cell-based oral tolerance protocol induces two subsets of Treg that systemically suppress antibody formation against FVIII or FIX, namely CD4^+^CD25^+^FoxP3^+^ and CD4^+^CD25^−^FoxP3^−^LAP^+^ Treg (124, 125, 127) (Figure 1). The latter are most robustly induced and produce IL-10 and TGF-β cytokines (125). LAP^+^ Tregs express latency-associated peptide (LAP) on the cell surface and suppress through a TGF-β dependent mechanism (131). Future work may show whether FoxP3^+^ and LAP^+^ Tregs have redundant or synergistic roles in oral tolerance induction to coagulation factors. Interestingly, there was also evidence for induction of type 1 Tregs in the lamina propria. These may locally contribute to tolerance induction through IL-10 expression. In fact, the oral tolerance mechanism was found to be IL-10 dependent, consistent with the notion that IL-10 is a key component of immune tolerance on mucosal interphases (125).

## Other Approaches

Maternal antigen transfer may offer hope for many genetic disorders that are diagnosed *in utero*. The advantages of this method are as follows: immaturity of immune system, absence of pre-existing antibodies, and early, antigen-specific tolerance induction (132, 133). A recent study in hemophilia A mice found that intravenous administration of Fc fusions of FVIII A2 and C2 domains resulted in effective antigen transfer into the developing fetal immune system via the neonatal Fc receptor (132). Moreover, the offspring of injected females showed robust tolerance to repeated challenges with FVIII when compared with offspring of non-treated mothers. A window for tolerance induction during gestation was identified, resulting in development of thymic-derived and peripherally induced antigen-specific Treg. A potential limitation for this approach is the large antigen dose that may be required for effective transfer to the fetus. Interestingly, Fc-conjugated FVIII is in advanced clinical development as a method to increase the half-life of FVIII during replacement therapy (12, 134). Building on the tolerogenic properties of Fc sequences, these molecules may be superior antigens for ITI in general (17).

In other studies, neonatal AAV gene transfer to hemophilia A mice directed sustained therapeutic FVIII expression (~5% of normal) and immunological unresponsiveness, with no antibodies being detected against AAV or FVIII. Mice also remained tolerant to a subsequent FVIII challenge in adjuvant, performed 8 weeks after gene transfer (135). Alternatively, activated platelets can serve as a vehicle to deliver FVIII to the site of vascular injury in patients with inhibitors. Transplantation of modified hematopoetic stem cells (HSCs) with FVIII under megakaryocyte-specific promoter restored hemostasis in hemophilia A mice with inhibitors (136). Here, FVIII is stored in α-granules, which protects FVIII from elimination by inhibitors, which would occur for FVIII that circulates in plasma. Activated platelets release FVIII containing α-granules at the site of vascular injury, thereby restoring hemostasis. Similarly, LV-modified autologous canine megakaryocytes (precursors of platelets) expressing FVIII in α-granules prevented bleeding episodes in hemophilia A dogs (137). These large animal studies further support that platelet-derived FVIII may potentially benefit hemophilia patients with inhibitors. This approach combines autologous HSC gene transfer with bone marrow conditioning and has also been shown to tolerize the transplant recipient animals to FVIII (138).

In conclusion, a large number of diverse innovative approaches to induce immune tolerance in the treatment of hemophilia A and thus prevent and/or reverse inhibitor formation to FVIII are currently in pre-clinical development (Table 1). Mechanistically, these primarily aim at tipping the balance of the immune response to Treg induction. Each approach has conceptual advantages and disadvantages, which have to be factored into decisions about translation studies in humans (Table 1). Since inhibitors form in young boys with hemophilia, an acceptable level of immune suppression or genetic manipulation would have to be determined for some of these approaches. Nonetheless, new superior technologies for antigen-specific ITI hold much promise to finally reduce inhibitor formation in the treatment of hemophilia A patients.

## Author Contributions

RH wrote parts of the article. He supervised generation of the article and edited it. AS and MB wrote parts of the article and generated the figures.

## Conflict of Interest Statement

RH is a member of the scientific advisory council of Applied Genetic Technologies Corporation and has received royalty payments from Spark Therapeutics. However, neither of these companies provided funding for the generation of this article or for the work described in this article. All other authors declare that the research was conducted in the absence of any commercial or financial relationships that could be construed as a potential conflict of interest.
